# The triglyceride-glucose index shows a stronger association with complex coronary artery disease than the ACEF score among young and middle-aged adults with aortic valve calcification

**DOI:** 10.3389/fcvm.2026.1753991

**Published:** 2026-04-01

**Authors:** Zhe Zhang, Zeyuan Fan, Yan Liu, Lei Dong, Bing Xiao

**Affiliations:** Department of Cardiology, Civil Aviation General Hospital, Beijing, China

**Keywords:** ACEF score, aortic valve calcification, coronary artery disease, SYNTAX score, TyG index

## Abstract

**Background:**

Aortic valve calcification (AVC) is an active pathophysiological process that shares metabolic risk factors with coronary artery disease (CAD). However, whether AVC modifies the associations between metabolic or physiological risk indices and coronary anatomical complexity remains uncertain, particularly in younger populations. This study aimed to determine if AVC alters the relationships of the triglyceride–glucose (TyG) index, a surrogate marker of insulin resistance, and the ACEF score, an indicator of physiological reserve, with anatomically complex CAD, and to compare their predictive performance in young and middle-aged adults.

**Methods:**

This cross-sectional study enrolled 326 consecutive patients aged 18–65 years who underwent coronary angiography. For the primary analysis of coronary lesion complexity, 66 patients with prior revascularization history were excluded, yielding a final analytic cohort of 260 patients. Anatomically complex CAD was defined as a SYNTAX score ≥23. AVC status, determined by echocardiography, was evaluated as a potential modifier. Multivariable logistic regression models were used to assess the associations of the TyG index and ACEF score with complex CAD, followed by formal testing for interaction between each index and AVC status. When significant interactions were identified, stratified analyses were performed. The discriminatory performance was evaluated using receiver operating characteristic (ROC) curve analysis in the analytic cohort and within AVC-stratified subgroups, with formal comparison of area under the curve (AUC) values between subgroups.

**Results:**

In the analytic cohort, both TyG index and ACEF score showed significant association with anatomically complex CAD. Formal interaction testing demonstrated that AVC status significantly modified these associations (*p* for interaction = 0.002). In patients with AVC, the TyG index emerged as an independent predictor of complex CAD (adjusted OR 3.82, 95% CI 1.69–8.63; AUC = 0.714), whereas the ACEF score was no longer significantly associated with lesion complexity. Conversely, in patients without AVC, the ACEF score remained a strong predictor of complex CAD, while the TyG index showed no significant predictive value. Notably, despite exhibiting more anatomically severe CAD, patients with AVC had a lower incidence of acute myocardial infarction compared with those without AVC (31.9% vs. 46.5%).

**Conclusion:**

Among young and middle-aged adults, AVC identifies a distinct phenotype of metabolically driven, anatomically complex CAD. In this context, the TyG index provides superior and phenotype-specific predictive value, supporting its integration into pathophysiology-informed risk stratification strategies for younger patients with CAD.

## 1 Introduction

Coronary artery disease (CAD) remains a leading cause of global mortality and morbidity. According to the Global Burden of Disease Study, an estimated 197 million individuals were living with CAD in 2019, with 9.14 million deaths attributable to this condition worldwide (1). Calcific aortic valve disease (CAVD), a pathological spectrum ranging from early aortic sclerosis to severe calcific aortic stenosis, represents one of the most common forms of valvular heart disease and can progress to cause significant left ventricular outflow tract obstruction (2). Aortic valve calcification (AVC), the hallmark of CAVD, is now recognized as an actively regulated biological process rather than a passive, age-related degeneration. This process is driven by valvular interstitial cell activation in response to inflammatory signaling, endothelial injury, and metabolic disturbances (3–5). Importantly, AVC shares numerous risk factors and pathophysiological pathways with CAD, including hypertension, dyslipidemia, diabetes, and chronic kidney disease (6). Critically, AVC has emerged as an independent predictor of major adverse cardiovascular events (MACE), including myocardial infarction and cardiovascular mortality (7–9), even after adjusting for traditional cardiovascular risk factors and the burden of coronary artery calcification itself. These findings suggest that AVC is not merely an epiphenomenon but rather a marker of heightened cardiovascular risk (9, 10). Despite the well-established prognostic significance of AVC in elderly populations, its clinical significance—particularly its relationship with coronary anatomical complexity—remains less clear in young and middle-aged adults.

Improved risk stratification in these younger demographic patients requires biomarkers and indices that reflect underlying pathophysiology. The triglyceride–glucose (TyG) index has emerged as a simple, reproducible surrogate marker of insulin resistance and has been shown to predict both the severity of CAD and the progression of atherosclerosis, thereby directly capturing metabolic dysregulation that contributes to both AVC and CAD development (11). In contrast, the ACEF score (Age, Creatinine, Ejection Fraction) efficiently captures a patient's physiological reserve and has demonstrated prognostic utility across a range of cardiovascular settings, including percutaneous coronary intervention (PCI) (12). Conceptually, the TyG index quantifies the intensity of the metabolic insult, whereas the ACEF score assesses the system's functional capacity to cope with it. However, it remains unclear whether the presence of AVC modifies the relationships between these markers and coronary anatomical complexity.

Accordingly, this study aimed to determine whether AVC status modifies the associations of the TyG index and ACEF score with complex CAD and, subsequently, to compare their predictive performance.

## 2 Materials and methods

### 2.1 Study population and design

This retrospective cross-sectional study was conducted at the Department of Cardiology, Civil Aviation General Hospital. A total of 326 consecutive patients aged 18–65 years who underwent coronary angiography between January 2021 and December 2023 were enrolled.

The primary objective was to determine whether AVC status modified the associations of the TyG index and ACEF score with coronary artery complexity, defined as SYNTAX score ≥23. AVC was diagnosed using a semi-quantitative grading system recommended by the European Association of Cardiovascular Imaging (EACVI), which assessed calcification based on the size and extent of echogenic foci on the aortic valve leaflets (13). Patients exhibiting Grade 2 or higher calcification (defined as the presence of isolated, small, calcified spots or greater) were included in the AVC group.

The exclusion criteria were as follows: 1) Congenital heart disease. 2) Significant native valvular heart disease other than AVC (e.g., moderate or severe stenosis or regurgitation of any valve). 3) Prior heart valve replacement surgery. 4) Long-term use of corticosteroids or immunosuppressive agents.

### 2.2 Data collection and definitions

Demographic and clinical data were retrospectively extracted from electronic medical records. The collected variables included:

**Baseline characteristics:** age, sex, and medical history (hypertension, type 2 diabetes, hyperlipidemia, current smoking).
**Laboratory parameters:** total cholesterol (TC), triglycerides (TG), high-density lipoprotein cholesterol (HDL-C), low-density lipoprotein cholesterol (LDL-C), serum creatinine (Cr), estimated glomerular filtration rate (eGFR), uric acid (UA), fasting blood glucose (FBG), glycated hemoglobin (HbA1c), and serum calcium (Ca) and phosphorus (P) levels.
**Calculated scores and functional data:** The TyG index was calculated using the formula: **Ln [fasting triglycerides (mg/dL)** **×** **fasting plasma glucose (mg/dL)/2]**.
**The ACEF score was calculated as:** Age (years)/Left Ventricular Ejection Fraction (LVEF, %) + 1 (if serum creatinine >176 μmol/L). LVEF was assessed by transthoracic echocardiography.
**Clinical diagnoses:** including acute myocardial infarction (AMI), unstable angina pectoris (UAP), stable angina pectoris (SAP), coronary arterial atherosclerosis (CAAS), and normal coronary arteries.

### 2.3 Imaging acquisition and analysis

#### 2.3.1 Echocardiography

Standard transthoracic echocardiography was performed using either a GE Vivid E9 or Philips Ultrasound system equipped with 2.0–5.0 MHz probes to comprehensively assess cardiac structure and function.

#### 2.3.2 Coronary angiography and lesion complexity assessment

Coronary angiography was performed for standard clinical indications using Siemens or Philips digital subtraction angiography systems. All angiograms were subsequently retrieved and reviewed for this study by experienced interventional cardiologists who were blinded to the study's specific objectives and echocardiographic findings.

Definition of Significant Stenosis and Diseased Vessels: significant coronary artery stenosis was defined as a luminal diameter narrowing of ≥50% in a major epicardial vessel. Based on this criterion, patients were classified as having left-main, single-vessel, double-vessel, or triple-vessel disease.

#### 2.3.3 SYNTAX score

The SYNTAX score was calculated for treatment-naïve patients [i.e., those without a history of PCI or coronary artery bypass grafting (CABG)] who had at least one lesion with ≥50% stenosis in a vessel with a diameter of ≥1.5 mm. The score was determined using the official online SYNTAX score calculator (https://syntaxscore.org/) by experienced interventional cardiologists. To minimize inter-observer variability, the SYNTAX scores were independently assessed by two interventional cardiologists, with discrepancies resolved by consensus or by a third senior investigator. Based on the score, patients were stratified into low (0–22), intermediate (23–32), or high (≥33) risk groups.

### 2.4 Statistical analysis

Statistical analyses were conducted using SPSS Statistics software (version 27.0; IBM Corp.). Continuous variables were expressed as mean ± standard deviation or median with interquartile range, based on their distribution. Categorical variables are expressed as numbers and percentages.

Group comparisons for continuous variables were performed using the independent samples *t*-test or the Mann–Whitney *U*-test, as appropriate. Categorical variables were compared using the Chi-square test or Fisher's exact test.

To evaluate effect modification by AVC, multivariable logistic regression model was constructed with complex CAD as the dependent variable. The TyG index and ACEF score were included as primary predictors and was adjusted for pre-specified covariate. Covariates for adjustment were selected based on established clinical knowledge and their well-recognized status as cardiovascular risk factors (age, sex, hypertension, diabetes mellitus, and current smoking), rather than through data-driven variable selection, to avoid overfitting. Interaction terms between AVC status and each predictor (TyG × AVC and ACEF × AVC) were subsequently introduced. A significant interaction (*p* < 0.05) was considered evidence of effect modification.

Stratified multivariable logistic regression analyses were then performed separately within the AVC and non-AVC subgroups, using the same set of adjustment covariates. The results of these regression analyses are presented as adjusted odds ratios (ORs) with 95% confidence intervals (CIs).

Further exploratory analyses, including univariable logistic regression for each variable of interest (age, sex, hypertension, diabetes mellitus, current smoking, ACEF score, and TyG index) and a multivariable logistic regression model including all these variables was constructed.

Receiver operating characteristic (ROC) curve analysis was used to assess discriminatory performance of the TyG index and ACEF score for predicting complex CAD. The area under the curve (AUC) was calculated, and optimal cut-off values were determined by maximizing the Youden index, with corresponding sensitivity and specificity reported. To compare the predictive performance between the AVC and non-AVC subgroups, the AUCs for each predictor were compared using the DeLong test.

A combined model incorporating both indices was also evaluated. Statistical significance was defined as a two-tailed *p*-value < 0.05.

In this exploratory study, we relied on consecutive enrollment rather than a pre-specified sample size. The sample size was determined by the available consecutive patients meeting the inclusion criteria during the study period. While this approach may limit the statistical power, particularly for subgroup analyses, the primary focus on interaction testing provides a robust framework for hypothesis generation. All subgroup findings should therefore be interpreted with appropriate caution.

## 3 Results

### 3.1 Baseline clinical characteristics of cohort stratified by complex coronary artery disease status

A total of 326 young and middle-aged patients who underwent coronary angiography were initially screened. For the primary analysis of native CAD complexity, 66 patients with a history of PCI or CABG were excluded, as the SYNTAX score is not applicable in these clinical contexts. The final analytic cohort therefore comprised 260 patients with evaluable SYNTAX scores.

As summarized in Table 1, 260 patients were stratified according to lesion complexity into an intermediate-to-high SYNTAX score group (≥23, *n* = 55) and a low SYNTAX score group (<23, *n* = 205). Overall, the two groups were comparable with respect to demographic characteristics and traditional cardiovascular risk factors. Although male sex was more prevalent in the SYNTAX ≥23 group (76.4% vs. 60.0%), this difference did not reach statistical significance (*p* = 0.118). No significant between-group differences were observed for age, smoking status, hypertension, diabetes mellitus, dyslipidemia, or the presence of AVC (all *p* > 0.05).

**Table 1:** Baseline characteristics of the analytic cohort stratified by Complex coronary artery disease (SYNTAX score ≥23 vs. <23).

Characteristic	SYNTAX ≥23 (*n* = 55)	SYNTAX <23 (*n* = 205)	*p*-value
Demographic Data			
Age, years	56.9 ± 6.5	57.2 ± 7.6	0.812
Age ≤40 (*n*, %)	1 (1.8)	8 (3.9)	1.000
Gender (Male, *n*, %)	42 (76.4)	123 (60.0)	0.118
Current Smoking (*n*, %)	28 (50.9)	112 (54.6)	0.623
Comorbidities (*n*, %)			
Hypertension	30 (54.5)	137 (67.6)	0.080
Diabetes Mellitus	26 (47.3)	73 (35.6)	0.071
Dyslipidemia	27 (49.1)	102 (53.2)	0.626
Laboratory Parameters			
TC (mmol/L)	4.83 ± 1.42	4.52 ± 1.19	0.131
TG (mmol/L)	2.01 (1.20–3.26)	1.74 (1.16–2.46)	0.116
HDL-C (mmol/L)	1.10 ± 0.26	1.21 ± 0.36	**0**.**020**
LDL-C (mmol/L)	3.00 ± 1.10	2.65 ± 0.88	**0**.**027**
TG/HDL-C ratio	1.78 (1.09–3.37)	1.45 (0.92–2.49)	0.051
LDL/HDL-C ratio	2.66 (2.30–3.41)	2.22 (1.93–2.64)	**0**.**017**
Cr (*μ*mol/L)	74.3 (62.2–93.8)	68.0 (56.9–79.8)	0.316
eGFR (mL/min/1.73 m^2^)	89.3 ± 28.1	92.6 ± 21.6	0.399
FBG (mmol/L)	8.10 ± 3.36	6.58 ± 2.40	**0**.**001**
HbA1c (%)	7.78 ± 2.17	6.72 ± 1.49	**<0**.**001**
Primary Study Metrics			
TyG Index	9.47 ± 0.76	9.12 ± 0.80	**0**.**017**
ACEF Score	1.17 ± 0.53	0.97 ± 0.29	**0**.**001**
AVC (*n*, %)	34 (61.8)	103 (50.2)	0.180
Clinical Presentation (*n*, %)			
Acute Myocardial Infarction	37 (67.3)	71 (34.6)	**<0**.**001**
Unstable Angina	18 (32.7)	76 (37.1)	
Stable Angina	0 (0)	40 (19.5)	
Coronary Atherosclerosis/Normal	0 (0)	18 (8.8)	
Echocardiographic Data			
LVEF (%)	60.0 (51.0–65.0)	64.0 (60.0–67.0)	**0**.**020**

SYNTAX scores were calculated for patients without a history of percutaneous coronary intervention or coronary artery bypass grafting.

TC, total cholesterol; TG, triglycerides; HDL-C, high-density lipoprotein cholesterol; LDL-C, low-density lipoprotein cholesterol; Cr, creatinine; eGFR, estimated glomerular filtration rate; FBG, fasting blood glucose; HbA1c, glycated hemoglobin; LVEF, left ventricular ejection fraction.

Bold values indicate statistical significance (*p* < 0.05).

In contrast, laboratory parameters demonstrated a substantially less favorable metabolic profile among patients with higher SYNTAX score. Compared with the low SYNTAX group, patients with SYNTAX ≥23 had significantly lower levels of HDL-C (1.10 ± 0.26 vs. 1.21 ± 0.36 mmol/L, *p* = 0.020) and higher levels of LDL-C (3.00 ± 1.10 vs. 2.65 ± 0.88 mmol/L, *p* = 0.027), FBG (8.10 ± 3.36 vs. 6.58 ± 2.40 mmol/L, *p* = 0.001), and HbA1c (7.78 ± 2.17% vs. 6.72 ± 1.49%, *p* < 0.001). Consistent with these findings, both the TyG index (9.47 ± 0.76 vs. 9.12 ± 0.80, *p* = 0.017) and ACEF score (1.17 ± 0.53 vs. 0.97 ± 0.29, *p* = 0.001) were significantly elevated in the SYNTAX ≥23 group.

Marked differences were also observed in clinical presentation. The distribution of clinical presentations also differed markedly between groups (*p* < 0.001), with AMI occurring far more frequently in patients with SYNTAX scores ≥23 than in those with lower scores (67.3% vs. 34.0%). Although LVEF remained within the normal range in both groups, echocardiographic assessment revealed a statistically lower median LVEF in the SYNTAX ≥23 group.

### 3.2 Association of the TyG index and ACEF score with high anatomical complexity (SYNTAX score ≥23) and effect modification by aortic valve calcification

We next evaluated the associations between the TyG index, the ACEF score, and the presence of complex CAD, defined as a SYNTAX score ≥23, using multivariable logistic regression analysis. To determine whether AVC modified these associations, interaction terms were incorporated into the adjusted models. The complete results are presented in Table 2.

**Table 2:** Interaction analysis and stratified associations of the TyG index and ACEF score with complex coronary artery disease (SYNTAX score ≥23).

Model and Predictor	Adjusted Odds Ratio (OR)	95% Confidence Interval (CI)	*p* value
Analytic Cohort (*n* = 260)			
TyG Index	1.80	1.05–3.09	**0**.**031**
ACEF Score	4.46	1.53–13.00	**0**.**006**
Interaction Terms			
TyG Index × AVC	–	–	**0**.**002**
ACEF Score × AVC	–	–	**0**.**010**
Stratified Model: AVC-Positive (*n* = 137)			
TyG Index	3.33	1.45–7.63	**0**.**005**
ACEF Score	1.72	0.56–5.32	0.345
Stratified Model: AVC-Negative (*n* = 123)			
TyG Index	1.23	0.62–2.43	0.562
ACEF Score	14.27	3.19–63.83	**<0**.**001**

All models were adjusted for age, sex, hypertension, diabetes mellitus, and current smoking status.

Bold values indicate statistical significance (*p* < 0.05).

#### 3.2.1 Initial analytic-cohort analysis

In the analytic cohort, a multivariable logistic regression model was constructed with mandatory adjustment for the established cardiovascular risk factors, including age, sex, hypertension, diabetes mellitus, and current smoking status. Within this framework, the ACEF score was a strong independent predictor of a SYNTAX score ≥23 [adjusted odds ratio [OR], 4.46; 95% confidence interval [CI], 1.53–13.00; *p* = 0.006]. The TyG index was also a significant predictor (adjusted OR, 1.80; 95% CI, 1.05–3.09; *p* = 0.031).

#### 3.2.2 Formal test for effect modification by aortic valve calcification

To formally assess whether the associations of the TyG index and ACEF score with complex coronary disease differed according to AVC status, interaction terms were added to the adjusted regression model. The analysis demonstrated strong statistical evidence for interaction. Both the TyG × AVC and ACEF × AVC terms were significant (*p* = 0.002 and *p* = 0.010, respectively). This means the predictive value of the TyG index and ACEF score is substantially modified by AVC status.

#### 3.2.3 Stratified multivariable analyses

**Figure 1:**
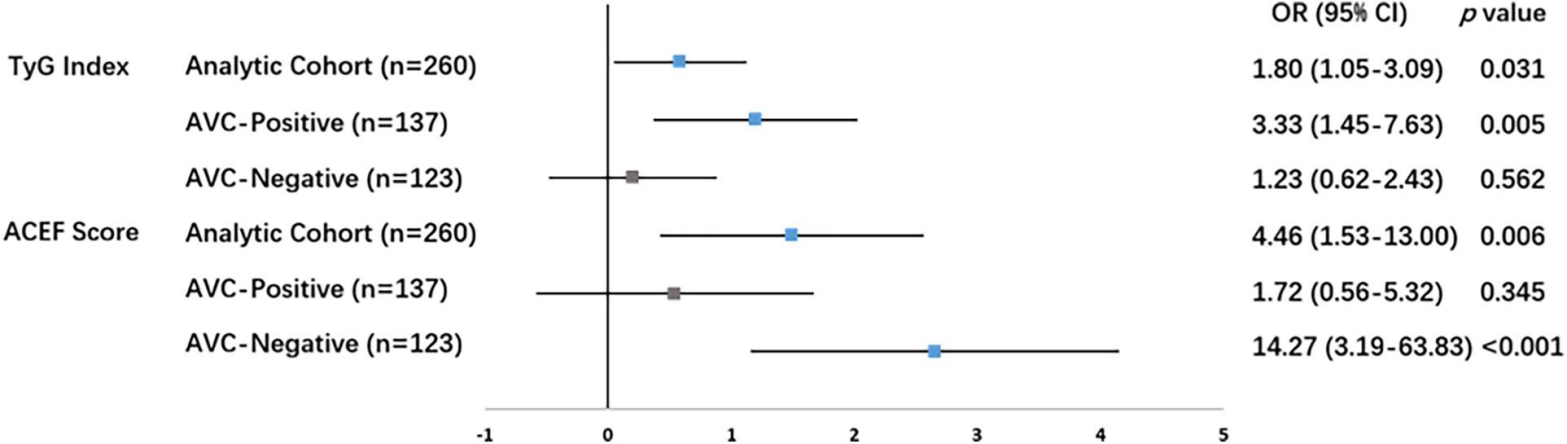
Forest plot of subgroup-specific associations stratified by aortic valve calcification status. The plot displays the natural logarithm of adjusted odds ratios (lnOR with 95% confidence intervals). The vertical reference line at lnOR = 0, which corresponds to an odds ratio of 1 (no association). All models were adjusted for age, sex, hypertension, diabetes mellitus, and current smoking. Blue solid squares denote statistically significant associations (*p* < 0.05).

Given the significant interaction effects, we performed stratified analyses within the AVC-positive and AVC-negative subgroups, applying identical covariate adjustment in both strata. These analyses revealed a strikingly divergent pattern of prediction, as illustrated in Table 2 and the corresponding forest plot (Figure 1).

Among patients with AVC, the TyG index was a strong and independent predictor of complex CAD (adjusted OR 3.33, 95% CI 1.45–7.63, *p* = 0.005), whereas the ACEF score showed no significant association (*p* = 0.345). Among patients without AVC, the TyG index was not predictive (*p* = 0.562), while the ACEF score demonstrated a very strong association with a SYNTAX score ≥23 (adjusted OR 14.27, 95% CI 3.19–63.83, *p* < 0.001).

#### 3.2.4 In-depth analysis of predictors within the AVC subgroup

To further clarify the determinants of coronary complexity in patients with AVC, a more detailed regression analysis was conducted within this subgroup. Univariable logistic regression was first performed for each variable, followed by construction of a multivariable model that simultaneously included age, sex, hypertension, diabetes mellitus, current smoking status, ACEF score, and TyG index (Table 3).

**Table 3:** Univariable and multivariable logistic regression analyses for predictors of a SYNTAX score ≥23 in the AVC subgroup (*n* = 137).

Variable	Univariable Analysis		Multivariable Analysis	
	OR (95% CI)	*p* value	Adjusted OR (95% CI)	*p* value
Age (per year)	0.92 (0.84–1.00)	**0**.**045**	1.00 (0.88–1.13)	0.952
Sex (Male)	0.52 (0.23–1.17)	0.114	0.60 (0.20–1.82)	0.366
Hypertension	0.44 (0.19–1.03)	0.058	0.40 (0.14–1.20)	0.102
Diabetes Mellitus	2.26 (1.00–5.12)	**0**.**050**	2.26 (0.75–6.83)	0.147
Current Smoking	0.85 (0.39–1.86)	0.692	0.49 (0.14–1.75)	0.273
ACEF Score	2.39 (0.82–6.92)	0.109	2.32 (0.62–8.63)	0.211
TyG Index	4.84 (2.03–11.57)	**<0**.**001**	4.77 (1.81–12.55)	**0**.**002**

OR, odds ratio; CI, confidence interval.

The multivariable model presented here is a comprehensive, fully-adjusted model that includes both the TyG index and the ACEF score alongside traditional risk factors. It is designed to assess the independent association of the TyG index within a model that accounts for ACEF score. For the primary analysis of effect modification by AVC status, which required separate evaluation of each score, please refer to Table 2.

Bold values indicate statistical significance (*p* < 0.05).

In this comprehensive model, the TyG index remained an independent predictor of higher SYNTAX scores (adjusted OR = 4.77, 95% CI 1.81–12.55, *p* = 0.002). In contrast, neither the ACEF score (*p* = 0.211) nor traditional cardiovascular risk factors achieved statistical significance.

### 3.3 Characterization of the subgroups with and without aortic valve calcification

#### 3.3.1 Baseline profile and clinical presentation in the full cohort

To comprehensively characterize the clinical phenotype associated with AVC, analyses were extended to the full consecutively enrolled cohort of 326 patients. Patients were classified into the AVC group (*n* = 182) and the non-AVC group (*n* = 144), with baseline characteristics detailed in Supplementary Table S1.

Patients with AVC were older and exhibited a higher prevalence of hypertension, diabetes mellitus, and dyslipidemia (all *p* < 0.001). Despite this adverse cardiometabolic profile, the AVC group included a lower proportion of males and current smokers. Laboratory findings were concordant with these clinical characteristics, with significantly higher FBG, HbA1c, and TG levels observed in the AVC group (all *p* < 0.05). Notably, a history of CABG was observed exclusively in the AVC group (6.6% vs. 0.0%, *p* < 0.001), whereas prior PCI was similarly prevalent in both groups (18.1% vs. 14.6%, *p* = 0.402).

**Figure 2:**
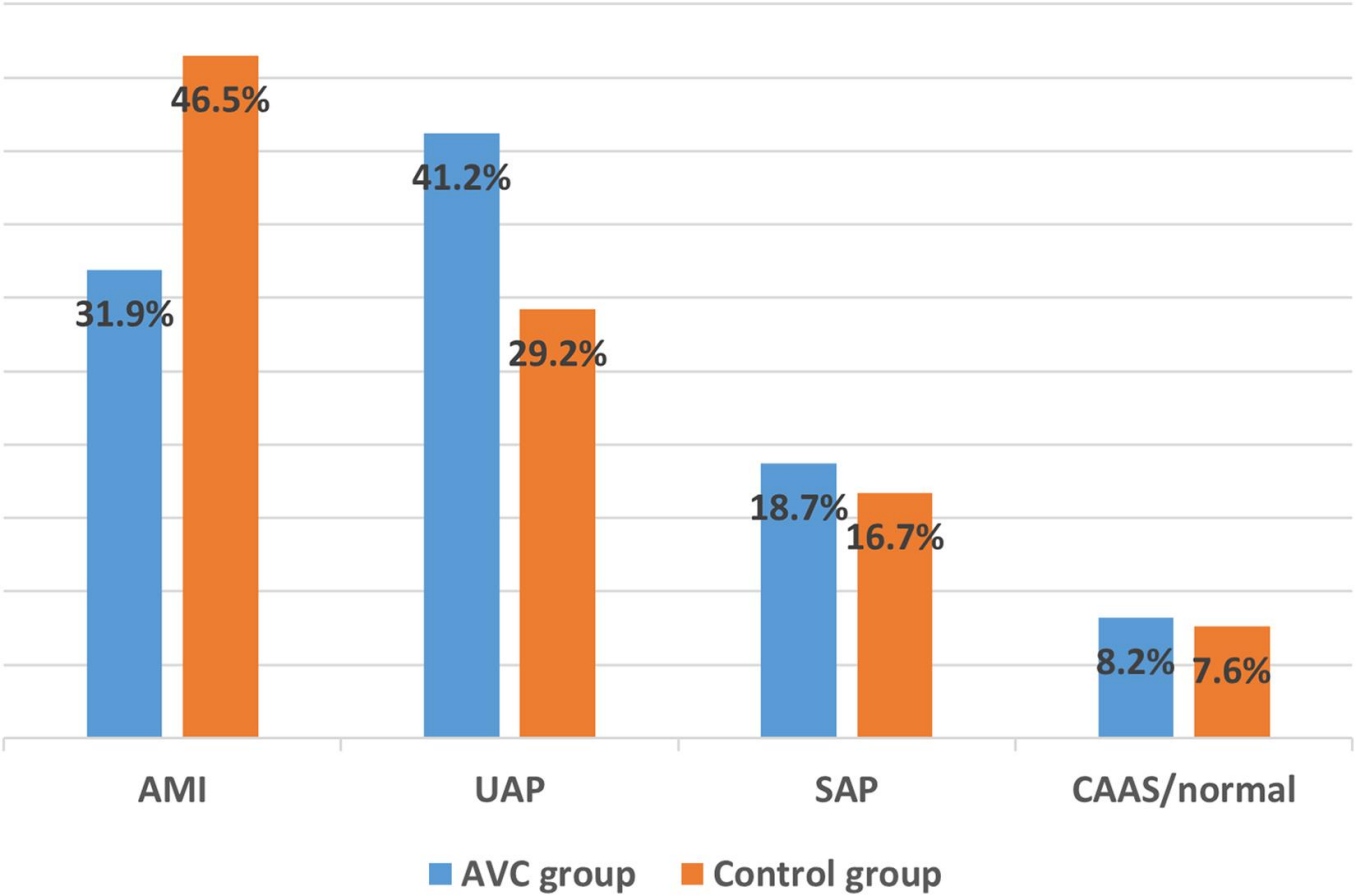
Clinical diagnoses at hospitalization in the full study cohort (*n* = 326), stratified by AVC Status. AMI, acute myocardial infarction; UAP, unstable angina pectoris; SAP, stable angina pectoris; CAAS, coronary arterial atherosclerosis.

The distribution of clinical diagnoses differed significantly between the two groups (*p* = 0.033), as shown in Figure 2. Although patients without AVC exhibited a more favorable metabolic profile, they were admitted with AMI more frequently than those with AVC (46.5% vs. 31.9%). Subtype analysis revealed that this difference was largely attributable to a substantially higher proportion of ST-segment elevation myocardial infarction (STEMI) in the non-AVC group (77.8% of their AMI cases vs. 55.2% in the AVC group, *p* = 0.022). Conversely, non-ST-segment elevation myocardial infarction (NSTEMI) was more common among those with AVC. UAP occurred more frequently in patients with AVC (41.2% vs. 29.2%), while the proportions of patients with SAP or CAAS or normal arteries were comparable between groups.

#### 3.3.2 Anatomical complexity of coronary lesions

For analyses focusing on native coronary anatomy and its relationship with the TyG index and ACEF score, attention was restricted to treatment-naïve analytic cohort of 260 patients, comprising 137 patients with AVC and 123 without.

Despite their lower incidence of AMI, patients with AVC exhibited a substantially more severe and complex pattern of CAD on angiographic evaluation. This was evident both in the distribution of diseased vessels and in SYNTAX score categories, as detailed in Table 4.

**Table 4:** Angiographic severity of coronary artery disease stratified by aortic valve calcification Status.

Characteristic	AVC Group (*n* = 137)	Non-AVC Group (*n* = 123)	*p* value
Number of Diseased Vessels, *n* (%)			
Left Main Disease	10 (7.3)	8 (5.6)	0.789
Single-Vessel Disease	27 (19.7)	42 (34.1)	**0**.**002**
Double-Vessel Disease	37 (27.0)	32 (26.1)	
Triple-Vessel Disease	63 (46.0)	45 (36.6)	
SYNTAX Score	10.0 (5.5–24.0)	11.0 (6.0–21.5)	0.722
SYNTAX Score Category, *n* (%)			
Low (0–22)	103 (75.2)	102 (82.9)	**0**.**028**
Intermediate (23–32)	25 (18.2)	20 (16.3)	
High (≥33)	9 (6.6)	1 (0.8)	

Bold values indicate statistical significance (*p* < 0.05).

Specifically, the AVC group had a lower prevalence of single-vessel disease (19.7% vs. 34.1%) and a higher prevalence of triple-vessel disease (46.0% vs. 36.6%), while the frequencies of left main and double-vessel disease were similar between groups.

The increased anatomical complexity in the AVC group was further substantiated by the SYNTAX score risk categorization, with a significantly greater proportion of these patients classified into the intermediate- or high-risk categories (24.8% vs. 17.1%). When considered alongside the lower incidence of AMI observed in this group, this dissociation between anatomical complexity and the clinical presentation of acute events highlights a notable paradox.

### 3.4 Discriminatory performance of the TyG index and ACEF score

**Figure 3:**
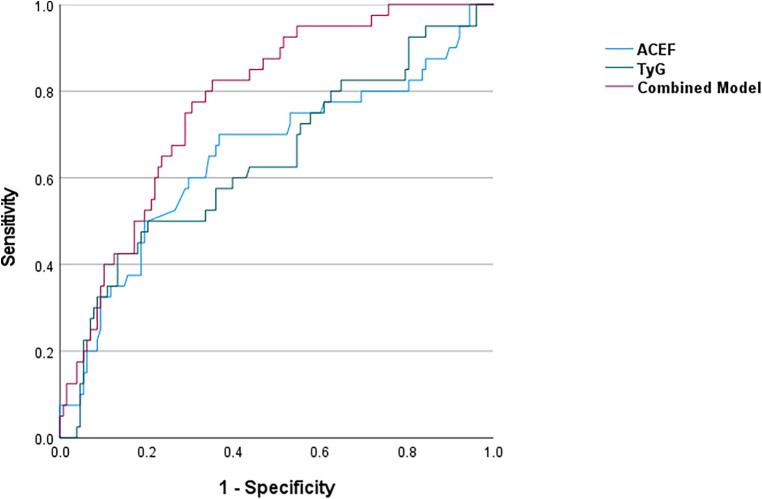
ROC curves of the TyG Index, ACEF score, and their combined model for predicting an intermediate-high SYNTAX score (≥23) in the analytic cohort (*n* = 260).

ROC curve analysis demonstrated that, within the analytic cohort, both the TyG index and the ACEF score had modest but statistically significant ability to discriminate complex CAD, defined as a SYNTAX ≥23), with AUCs of 0.649 and 0.628, respectively. The combined model incorporating both indices yielded a higher AUC (0.723), indicating improved discriminatory performance (Table 5, Figure 3).

**Table 5:** Predictive performance of the TyG index, ACEF score, and their combined model for complex CAD (SYNTAX score ≥23) in the analytic cohort and stratified by aortic valve calcification status.

Predictor	Analytic Cohort (*n*=260)		AVC Group (*n*=137)		Non-AVC Group (*n*=123)		AUC Comparison (AVC vs. Non-AVC)	
	AUC (95% CI);	*p* value	AUC (95% CI);	*p* value	AUC (95% CI);	*p* value	*Δ*AUC (95% CI);	*p* value
TyG Index	0.649 (0.576–0.722);	**<0** **.** **001**	0.714 (0.611–0.817);	**<0** **.** **001**	0.503 (0.376–0.630);	0.975	0.220 (0.003 to 0.437);	**0** **.** **047**
ACEF Score	0.628 (0.553–0.702);	**0** **.** **012**	0.585 (0.462–0.708);	0.187	0.616 (0.488–0.744);	0.079	−0.042 (−0.282 to 0.198);	0.733
Combined Model	0.723 (0.654–0.792);	**<0** **.** **001**	0.722 (0.620–0.823);	**<0** **.** **001**	0.736 (0.624–0.848);	**0** **.** **002**	0.053 (−0.124 to 0.231);	0.554

Data are presented as AUC with 95% CI and *p*-value (testing against AUC = 0.5). The combined model is the predicted probability from a multivariable logistic regression model. The AUC difference (*Δ*AUC) between AVC and Non-AVC groups was compared using the DeLong test (*p*-value for interaction).

Bold values indicate statistical significance (*p* < 0.05).

**Figure 4:**
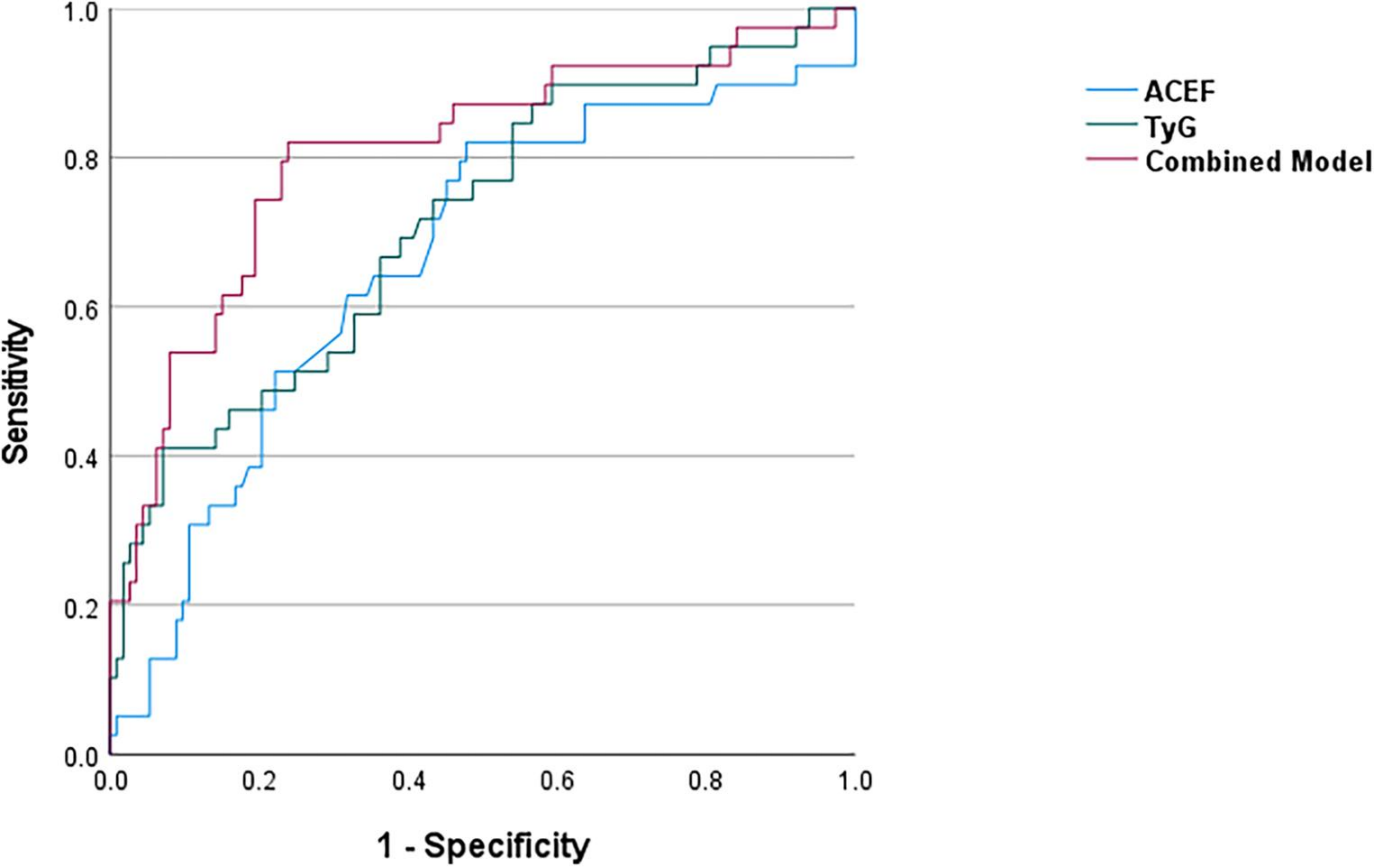
ROC curves of the TyG Index, ACEF score, and their combined model for predicting an intermediate-high SYNTAX score (≥23) in the AVC subgroup (*n* = 137).

Stratified ROC analyses revealed a clear divergence in predictive performance according to AVC status (Figure 4). In patients with AVC, the TyG index demonstrated good discriminatory ability (AUC = 0.714), whereas the ACEF score showed no significant predictive value (AUC = 0.585, *p* = 0.187). Conversely, in patients without AVC, the TyG index performed no predictive value (AUC = 0.503), whereas the ACEF score exhibited a non-significant trend toward discrimination (AUC = 0.616, *p* = 0.079).

Formal comparison using the DeLong test confirmed that AVC status significantly modified the predictive performance of the TyG index (ΔAUC = 0.220, *p* = 0.047), as reflected by a significant difference in AUCs between AVC strata. No significant effect modification was observed for the ACEF score (*p* = 0.733) or the combined model (*p* = 0.554).

From a clinical perspective, the optimal TyG index cut-off in the analytic cohort was 9.24, corresponding to a Youden's index of 0.260 (65.5% sensitivity, 60.5% specificity). This cut-off was higher (9.33) and demonstrated better discriminatory power in the AVC subgroup, with a Youden's index of 0.381 (71.4% sensitivity, 66.7% specificity). The optimal ACEF score cut-off was 1.05 (Youden's index = 0.223; 56.4% sensitivity, 65.9% specificity).

## 4 Discussion

The principal finding of this cross-sectional study is that AVC significantly modifies the relationship between metabolic markers and coronary anatomical complexity in young and middle-aged adults. Formal interaction analysis demonstrated that AVC acts as a true effect modifier in the association between the TyG index and complex CAD, defined by a SYNTAX score ≥23. Two central insights emerge from these findings. First, within the AVC subgroup, the TyG index—a surrogate marker of insulin resistance—showed a strong and independent association with complex coronary anatomy, whereas the ACEF score, a composite measure of physiological reserve, did not retain independent predictive value. Second, a paradoxical dissociation was observed whereby patients with AVC exhibited more anatomically severe CAD yet experienced a lower incidence of AMI compared with their non-AVC counterparts. Together, these observations support the hypothesis that AVC identifies a distinct, metabolically driven atherosclerotic phenotype and underscore the clinical relevance of incorporating pathophysiology-based markers, such as the TyG index, into contemporary risk stratification strategies beyond chronological age and conventional physiological scoring systems.

The robust association between the TyG index and SYNTAX scores within the AVC subgroup points toward a shared pathophysiological substrate characterized by metabolic dysregulation, which may simultaneously promote valvular and coronary atherosclerosis. Consistent with this framework, baseline characteristics in our cohort revealed a higher prevalence of diabetes and dyslipidemia among patients with AVC, aligning with the established understanding that CAVD is an active, biologically regulated phenomenon influenced by metabolic factors (2, 3). Insulin resistance, as reflected by the TyG index (11), serves as a key driver of this process, promoting endothelial dysfunction, chronic inflammation, and osteogenic differentiation of valvular interstitial cells (3, 4). We further propose that the same metabolic milieu also accelerates coronary atherosclerosis by fostering a pro-inflammatory, pro-proliferative state within the vascular wall (14, 15). Such a systemic dysmetabolic state provides a plausible mechanistic explanation for the increased prevalence of complex, calcified, and multi-vessel lesions observed in our AVC subgroup (16). Within this context, the TyG index may represent an integrative marker that captures the cumulative burden of metabolic risk, offering a practical means to identify patients with AVC at heightened risk for anatomically severe CAD. The exclusive occurrence of prior CABG in the AVC cohort further supports the notion that this phenotype is associated with historically more complex and advanced CAD.

One of the most clinically intriguing findings of our study is the dissociation between anatomical severity and clinical presentation: Despite having more complex CAD, patients with AVC experienced a lower incidence of AMI, particularly STEMI, than patients without AVC. This apparent paradox may be explained by the presence of divergent atherosclerotic phenotypes across the two groups. In younger, relatively metabolically healthier individuals without AVC, coronary disease may be characterized by a higher proportion of lipid-rich plaques with large necrotic cores and thin fibrous caps, rendering them susceptible to sudden rupture upon acute inflammation, leading to occlusive thrombosis and the classic presentation of STEMI. This paradigm aligns with studies demonstrating a high prevalence of vulnerable, non-calcified plaques in young patients presenting with first-onset AMI (17, 18). Conversely, patients with AVC may be predisposed to a diffuse, pro-calcific disease process, resulting in widespread, heavily calcified, and often multi-vessel disease. Although anatomically complex, such plaques tend to be more stable, with thicker fibrous caps, and are therefore less prone to spontaneous rupture. Clinically, this phenotype may manifest more frequently as ischemia, unstable angina, or NSTEMI, driven by progressive stenosis or plaque erosion (5). Chronic insulin resistance fuels inflammation, which in turn promote both vascular and valvular calcification (19). It is, therefore, plausible that in patients with AVC, this pro-calcific environment favors the development of densely calcified coronary plaques. While this contributes to greater anatomical severity, such calcified plaques are generally more stable and less likely to rupture (20). This concept of a “calcified-stable” phenotype is further supported by broader evidence linking inflammatory activity to both coronary calcification and plaque stability (21). Thus, AVC may represent a readily detectable clinical marker of an underlying “chronic metabolic” type of atherosclerosis.

The limited predictive performance of the ACEF score within our AVC cohort provides critical insight into the distinct nature of cardiovascular risk in this population. The ACEF score, which integrates age, creatinine, and LVEF, is a validated measure of physiological reserve and is highly effective in older, multimorbid populations (12). However, in our comparatively young and middle-aged population, the restricted age range (all ≤65 years) and preserved left ventricular systolic function (median LVEF >59% in both groups) substantially constrained its discriminatory power. This fundamental limitation underscores that in the context of AVC, the “metabolic age” of the vascular system, as quantified by the TyG index, may be a more potent and proximate determinant of anatomical disease complexity than the “chronological age” captured by the ACEF score. Combining the TyG index with the ACEF score significantly enhanced risk discrimination in the overall cohort (AUC = 0.723, *p* < 0.001). This integrated approach offers a more robust tool for assessing risk in the broader population of young and middle-aged patients undergoing angiography.

These findings have direct implications for risk stratification and clinical decision-making. In young and middle-aged adults with evidence of AVC, calculation of the simple and cost-effective TyG index may help identify individuals at high risk for underlying complex CAD, thereby justifying a lower threshold for advanced vascular imaging or invasive angiography. This strategy is supported by both our results and accumulating evidence linking a higher TyG index to adverse cardiovascular outcomes in CAD populations (22). Conversely, the absence of AVC, particularly in younger patients presenting with acute symptoms, alerts clinicians to a different risk profile—one characterized by potentially vulnerable, rupture-prone plaques. Together, these observations support a “dual-phenotype” model that advocates for a refined, pathophysiology-informed strategy that moves beyond uniform application of conventional scoring systems.

Our study has some limitations. First, the cross-sectional design precludes causal inference regarding the observed associations. Second, as a single-center, retrospective analysis, the study is vulnerable to selection bias and unmeasured confounding, and the findings require confirmation in larger, prospective, multi-center cohorts. Third, in terms of our analytical framework, the modest sample size—particularly within the AVC subgroup—limited the statistical power of multivariable and subgroup analyses, as reflected by the wide confidence intervals of some estimates, underscoring the exploratory nature of these findings. Fourth, AVC was diagnosed and graded using echocardiography, which is clinically practical and guideline-recommended (14), is less sensitive than cardiac computed tomography for detecting early or micro-calcification (7, 8). This limitation may have resulted in misclassification of patients with subtle calcification into the non-AVC group. Finally, the mechanistic interpretation of divergent plaque phenotypes remains hypothetical, as direct assessments of plaque morphology or inflammatory biomarkers were not available in this study.

## 5 Conclusion

In conclusion, this study demonstrates that the presence of AVC in younger and middle-aged adults identifies a unique, high-risk coronary phenotype characterized by metabolic dysregulation and increased anatomical complexity, for which the TyG index emerges as a highly relevant predictor. The counterintuitive observation of a higher incidence of AMI among non-AVC patients highlights a critical distinction in underlying plaque biology between these groups. Collectively, these findings support a paradigm shift toward a pathophysiology-driven, phenotype-specific approach to cardiovascular risk assessment that integrates AVC status and metabolic markers rather than relying solely on traditional, age-centered risk models.

Future prospective, multi-center studies are warranted to validate the prognostic utility of the TyG index in patients with AVC and to directly characterize the proposed plaque phenotypes using advanced imaging techniques, such as coronary computed tomography angiography and intravascular ultrasound.

## Data Availability

The raw data supporting the conclusions of this article will be made available by the authors, without undue reservation.

## References

[1] Safiri S Karamzad N Singh K Carson-Chahhoud K Adams C Nejadghaderi SA Burden of ischemic heart disease and its attributable risk factors in 204 countries and territories, 1990–2019. Eur J Prev Cardiol. (2022) 29(2):420–31. 10.1093/eurjpc/zwab213 34922374

[2] Rajamannan NM Evans FJ Aikawa E Grande-Allen KJ Demer LL Heistad DD Calcific aortic valve disease: not simply a degenerative process: a review and agenda for research from the national heart and lung and blood institute aortic stenosis working group *Executive Summary: calcific Aortic Valve Disease—2011 Update*. Circulation. (2011) 124(16):1783–91. 10.1161/CIRCULATIONAHA.110.006767 22007101 PMC3306614

[3] Rattazzi M Bertacco E Iop L D'Andrea S Puato M Buso G Extracellular pyrophosphate is reduced in aortic interstitial valve cells acquiring a calcifying profile: implications for aortic valve calcification. Atherosclerosis. (2014) 237(2):568–76. 10.1016/j.atherosclerosis.2014.10.027 25463090

[4] Driscoll K Cruz AD Butcher JT. Inflammatory and biomechanical drivers of endothelial-interstitial interactions in calcific aortic valve disease. Circ Res. (2021) 128(9):1344–70. 10.1161/CIRCRESAHA.121.318011 33914601 PMC8519486

[5] Voisine M Hervault M Shen M Boilard A Filion B Rosa M Age, sex, and valve phenotype differences in fibro-calcific remodeling of calcified aortic valve. J Am Heart Assoc. (2020) 9(10):e015610. 10.1161/JAHA.119.015610 32384012 PMC7660864

[6] Ferreira-González I Pinar-Sopena J Ribera A Marsal JR Cascant P González-Alujas T Prevalence of calcific aortic valve disease in the elderly and associated risk factors: a population-based study in a Mediterranean area. Eur J Prev Cardiol. (2013) 20(6):1022–30. 10.1177/2047487312451238 22679252

[7] Owens DS Budoff MJ Katz R Takasu J Shavelle DM Carr JJ Aortic valve calcium independently predicts coronary and cardiovascular events in a primary prevention population. JACC Cardiovasc Imaging. (2012) 5(6):619–25. 10.1016/j.jcmg.2011.12.023 22698532 PMC3376353

[8] Di Minno MND Di Minno A Ambrosino P Songia P Pepi M Tremoli E Cardiovascular morbidity and mortality in patients with aortic valve sclerosis: a systematic review and meta-analysis. Int J Cardiol. (2018) 260:138–44. 10.1016/j.ijcard.2018.01.054 29622430

[9] Christensen JL Tan S Chung HE Ghosalkar DS Qureshi R Yu W Aortic valve calcification predicts all-cause mortality independent of coronary calcification and severe stenosis. Atherosclerosis. (2020) 307:16–20. 10.1016/j.atherosclerosis.2020.06.019 32702536 PMC7583087

[10] Williams MC Massera D Moss AJ Bing R Bularga A Adamson PD Prevalence and clinical implications of valvular calcification on coronary computed tomography angiography. Eur Heart J—Cardiovasc Imaging. (2021) 22(3):262–70. 10.1093/ehjci/jeaa263 33306104 PMC7899264

[11] Liu Y Zhu B Zhou W Du Y Qi D Wang C Triglyceride–glucose index as a marker of adverse cardiovascular prognosis in patients with coronary heart disease and hypertension. Cardiovasc Diabetol. (2023) 22(1):133. 10.1186/s12933-023-01866-9 37296406 PMC10257289

[12] Wykrzykowska JJ Garg S Onuma Y de Vries T Goedhart D Morel M-A Value of age, creatinine, and ejection fraction (ACEF score) in assessing risk in patients undergoing percutaneous coronary interventions in the “all-comers” LEADERS trial. Circ Cardiovasc Interv. (2011) 4(1):47–56. 10.1161/CIRCINTERVENTIONS.110.958389 21205944

[13] Lancellotti P Pibarot P Chambers J La Canna G Pepi M Dulgheru R Multi-modality imaging assessment of native valvular regurgitation: an EACVI and ESC council of valvular heart disease position paper. Eur Heart J—Cardiovasc Imaging. (2022) 23(5):e171–232. 10.1093/ehjci/jeab253 35292799

[14] Hao QY Gao JW Zeng YH Zhang S-L Xiong Z-C Yang P-Z Roles of triglyceride-glucose index in aortic valve calcification progression: a prospective and Mendelian randomization analysis. Clin Radiol. (2025) 84:106860. 10.1016/j.crad.2025.106860 40106977

[15] Wang P Zeng Y Wang L Jiang Y Shen J Jin F Association of TyG index with aortic valve calcification in valvular heart disease patients. Postgrad Med J. (2024) 100(1190):917–24. 10.1093/postmj/qgae085 39001659

[16] Beckman JA Creager MA Libby P. Diabetes and atherosclerosis: epidemiology, pathophysiology, and management. JAMA. (2002) 287(19):2570. 10.1001/jama.287.19.2570 12020339

[17] Juan-Salvadores P Olivas-Medina D De La Torre Fonseca LM Veiga C Campanioni S Caamaño Isorna F Clinical features and long-term outcomes in patients under 35 years with coronary artery disease: nested case–control study. Rev Port Cardiol. (2025) 44(1):13–21. 10.1016/j.repc.2024.06.004 39227005

[18] Zasada W Bobrowska B Plens K Dziewierz A Siudak Z Surdacki A Acute myocardial infarction in young patients. Kardiol Pol. (2021) 79(10):1093–8. 10.33963/KP.a2021.0099 34472075

[19] ChengG LiuY XingY ShiZ FaragMA JinS Lactylation at the metabolic-epigenetic interface in cardiovascular diseases: context-dependent mechanisms and translational roadmap. J Adv Res. (2026):S209012322600007X. 10.1016/j.jare.2026.01.00741490839

[20] Fountas P Gialeli C Thorsen NW Acoba D Sun J Gamon LF Elevated asporin expression in human atherosclerotic plaques promotes their stability and reduces the risk for cardiovascular events. Cardiovasc Res. (2026):cvag015. 10.1093/cvr/cvag015 PMC13019687 41557654

[21] Hao Z. Impact of inflammatory factors on coronary CT calcification score: correlation with plaque stability and evaluation of diagnostic efficiency. Am J Transl Res. (2025) 17(11):9022–31. 10.62347/BDUM2830 41415080 PMC12709308

[22] Zhang C Li M Liu L Zhong Y Xie Y Liao B Triglyceride-glucose index as a novel predictor of major adverse cardiovascular events in patients with coronary revascularization: a meta-analysis of cohort studies. Ann Med. (2026) 58(1):2607796. 10.1080/07853890.2025.2607796 41454630 PMC12777766

