# Supplementary material for: The triglyceride-glucose index shows a stronger association with complex coronary artery disease than the ACEF score among young and middle-aged adults with aortic valve calcification
Source: Front Cardiovasc Med. 2026 Apr 1;13:1753991. doi: 10.3389/fcvm.2026.1753991 (PMC13079005; doi:10.3389/fcvm.2026.1753991)
Supplement: Supplementary file 1 [file Table1.docx]

**Supplementary Table S1 Baseline Characteristics of the Full Study Cohort (n=326) Stratified by Aortic Valve Calcification Status**

| Characteristic | AVC Group  (n=182) | Non-AVC Group  (n=144) | *p* value |
| --- | --- | --- | --- |
| Demographic Data |  |  |  |
| Age, years | 60.1 ± 4.2 | 53.4 ± 8.9 | <0.001 |
| Gender (n, %) |  |  |  |
| Male | 102 (56.0) | 114 (79.2) | ＜0.001 |
| Female | 80 (44.0) | 30 (20.8) |  |
| Current Smoking (n, %) | 89 (48.9) | 90 (62.5) | 0.031 |
| Medical History (n, %) |  |  |  |
| CAD | 53 (29.1) | 32 (22.2) | 0.123 |
| History of PCI | 33(18.1) | 21(14.6) | 0.402 |
| History of CABG | 12(6.6) | 0(0.0) | ＜0.001 |
| Hypertension | 136 (74.7) | 83 (57.6) | ＜0.001 |
| Diabetes Mellitus | 94 (51.6) | 35 (24.3) | ＜0.001 |
| Dyslipidemia | 113 (62.1) | 63 (43.8) | ＜0.001 |
| Laboratory Parameter |  |  |  |
| TC (mmol/L) | 4.44±1.18 | 4.61±1.33 | 0.201 |
| TG (mmol/L) | 1.82(1.22-2.48) | 1.67(1.17-2.90) | 0.024 |
| HDL-C (mmol/L) | 1.19±0.33 | 1.18±0.35 | 0.816 |
| LDL-C (mmol/L) | 2.60±0.91 | 2.74±0.98 | 0.174 |
| TG/HDL-C ratio | 1.82(0.94-2.42) | 2.68(0.93-2.83) | 0.019 |
| LDL/HDL-C ratio | 2.28±0.91 | 2.53±1.21 | 0.029 |
| Cr (μmol/L) | 71.4(57.9-85.5) | 67.8(56.9-79.5) | 0.289 |
| eGFR (ml/min/1.73m^2^) | 88.0(81.6-101.0) | 96.2(90.6-108.0) | <0.001 |
| UA | 352.2±108.6 | 367.1±98.8 | 0.166 |
| FBG (mmol/L) | 7.1(5.3-8.4) | 6.4(5.0-6.6) | 0.030 |
| HbA1c | 7.1(6.0-7.8) | 6.5(5.7-6.8) | <0.001 |
| Ca (mmol/L) | 2.27±0.14 | 2.29±0.12 | 0.194 |
| P (mmol/L) | 1.13±0.29 | 1.11±0.25 | 0.230 |
| Cardiac Function |  |  |  |
| LVEF (%) | 59(55-66) | 61.5(60-67) | 0.044 |

**Abbreviations:** TC, total cholesterol; TG, triglycerides; HDL-C, high-density lipoprotein cholesterol; LDL-C, low-density lipoprotein cholesterol; Cr, creatinine; eGFR, estimated glomerular filtration rate; UA, uric acid; FBG, fasting blood glucose; HbA1c, glycated hemoglobin; Ca, calcium; P, phosphorus; LVEF, left ventricular ejection fraction.
